# First person – Karen Boschen

**DOI:** 10.1242/dmm.049131

**Published:** 2021-06-17

## Abstract

First Person is a series of interviews with the first authors of a selection of papers published in Disease Models & Mechanisms, helping early-career researchers promote themselves alongside their papers. Karen Boschen is first author on ‘ Transcriptomic analyses of gastrulation-stage mouse embryos with differential susceptibility to alcohol’, published in DMM. Karen is a postdoctoral trainee in the lab of Dr Scott Parnell at The University of North Carolina at Chapel Hill, Chapel Hill, NC, USA, investigating the cellular mechanisms of prenatal alcohol exposure, and genetic factors that influence risk and resiliency to developing alcohol-related birth defects.


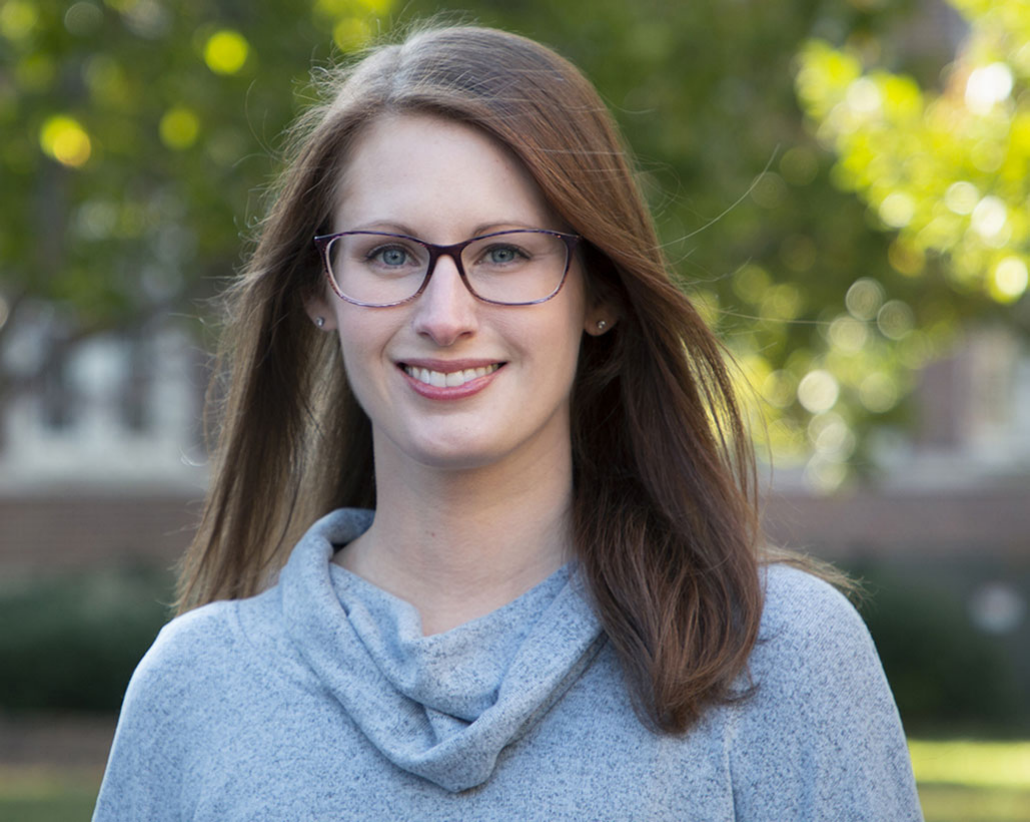


**Karen Boschen**

**How would you explain the main findings of your paper to non-scientific family and friends?**

Foetal alcohol spectrum disorders (FASDs) is a term referring to a group of conditions resulting from exposure to alcohol in the womb. FASDs are a serious public health concern, affecting ∼5% of live births in the USA each year. Consequences can include serious birth defects affecting the face and brain, difficulties with learning and impulse control, and long-term cognitive, behavioural and memory impairments. An ongoing question in the FASD field asks why some children exposed to alcohol during pregnancy develop significant physical and cognitive deficits whereas others are relatively unaffected. The goal of our study was to investigate the contribution of genetics to the development of prenatal alcohol-induced birth defects using a well-characterized mouse model of FASD. Mice provide a good model system to study how alcohol affects foetal development, allowing researchers to determine sensitive periods and investigate how other internal and external factors, such as genetics, mediate the severity of alcohol damage. In this study, we used two mouse strains that are mostly genetically similar to one another but differ in their susceptibility to prenatal alcohol exposure: one strain is very sensitive and has a higher rate of alcohol-induced craniofacial defects compared to the other strain, which is more resistant. We found that the sensitive strain had increased expression of genes related to immune molecule signalling and inflammation at baseline, before alcohol was administered. After alcohol exposure, the sensitive strain had more genes that were dysregulated compared to the resistant strain. In addition, the gene pathways targeted by alcohol in the sensitive strain were more likely to be related to cell death and craniofacial development. These data show that relatively small amounts of genetic variation can cause significant differences in the cellular response to a teratogen, such as alcohol, and in long-term outcomes, such as severity of birth defects. In addition, this dataset has provided useful information related to normal gene expression patterns in the gastrulation-stage mouse embryo, which can be explored in a web tool we built for gene-by-gene exploration of the data (http://parnell-lab.med.unc.edu/Embryo-Transcriptomics/).

“The web tool we created is a valuable resource for developmental biologists and toxicologists […]”

**What are the potential implications of these results for your field of research?**

Our results provide new information about normal gene expression patterns during gastrulation in two commonly used mouse substrains, C57BL/6J and C57BL/6NHsd. The web tool we created is a valuable resource for developmental biologists and toxicologists that study gene expression during this developmental window. In addition, this study will contribute to the discovery of candidate genes that may modify susceptibility to prenatal alcohol exposure that can be validated in human patients.

**What are the main advantages and drawbacks of the model system you have used as it relates to the disease you are investigating?**

There are two main advantages to using the mouse as a model system to study FASD. First, the mouse goes through the same developmental stages as human embryos do, allowing us to target very specific developmental events with our alcohol exposure. Using this approach, researchers have determined that administration of alcohol during gastrulation (embryonic day 7 in mice; third week of pregnancy in humans) causes the characteristic craniofacial features associated with the most severe FASD, foetal alcohol syndrome. Alcohol exposure during other developmental periods has significant impacts on the development of other organs, such as the brain, heart or limbs, but does not cause the same craniofacial birth defects. Second, studying the contribution of genetics is very easy in mice, using either inbred mouse strains like we did in this paper, or transgenic mice that have mutations in single genes. However, mice are not humans, and are one tool that we can use in the discovery of the cellular mechanisms of prenatal alcohol exposure.

**What has surprised you the most while conducting your research?**

**Figure:**
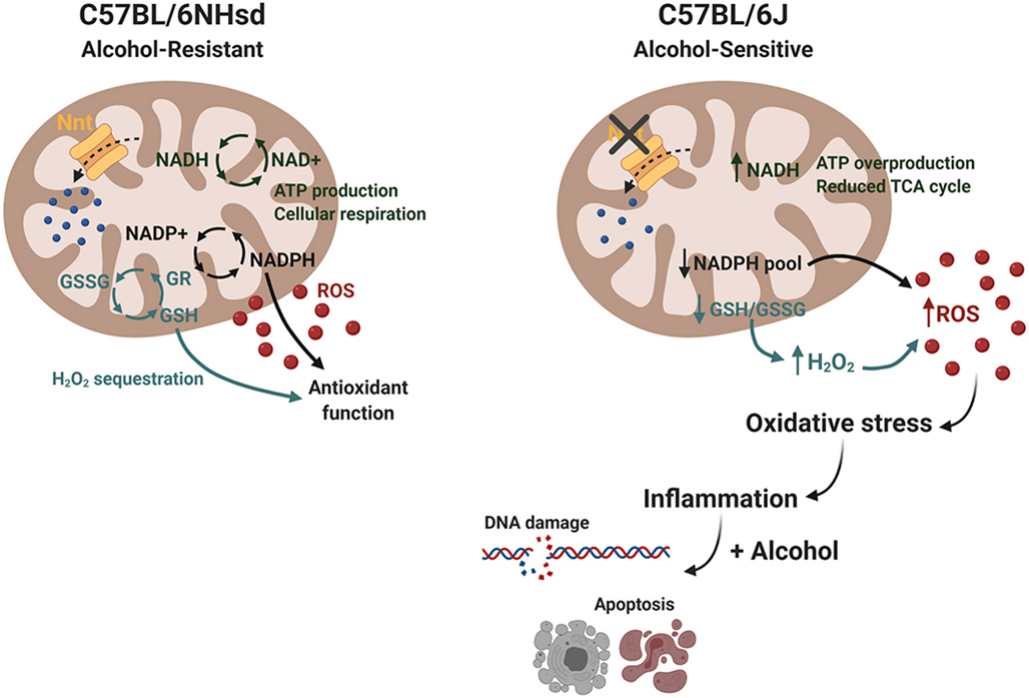
**A schematic representing a hypothetical mechanism contributing to the differing alcohol sensitivity observed between the C57BL/6J (sensitive) and C57BL/6N (resistant) mouse strains.** The known *Nnt* mutation in the C57BL/6J strain could affect reactive oxygen species breakdown in the mitochondria, leading to higher baseline oxidative stress and inflammation. In the presence of prenatal alcohol, this increased baseline expression of immune signalling genes would cause the 6J embryos to undergo increased apoptosis and DNA damage, ultimately resulting in more severe craniofacial and central nervous system anomalies. GR, glutathione reductase; GSSG, glutathione disulfide; GSH, glutathione; NAD+/NADH, nicotinamide adenine dinucleotide (+ hydrogen); NADP+/NADPH, nicotinamide adenine dinucleotide phosphate; Nnt, nicotinamide nucleotide transhydrogenase.

The two mouse strains we used in this paper, the C57BL/6J and C57BL/6NHsd strains, are both commonly used, genetically similar inbred strains. Previous work has shown that the 6J strain is significantly more likely to develop craniofacial birth defects after prenatal alcohol compared to the 6N strain. I was surprised at how few genes differed between the strains at baseline – only 80 genes! However, these small differences end up having a big impact both 6-12 h after alcohol exposure, as we assessed in this study, and later in development, when the physical defects caused by alcohol become apparent.

“[…] few genes differed between the strains at baseline – only 80 genes! However, these small differences end up having a big impact […]”

**Describe what you think is the most significant challenge impacting your research at this time and how will this be addressed over the next 10 years?**

Understanding the determinants that can influence a foetus's risk to serious harm from prenatal alcohol exposure is currently a major focus of our field. Both biological factors such as genetics, foetal sex, maternal metabolism and health status, and environmental influences, such as diet, poly-drug exposure and stress, are important areas of interest for FASD researchers over the next decade. Designing experiments that address how these factors, either alone or in combination, affect the alcohol-exposed embryo will be critical to improving outcomes in individuals with FASD.

**What changes do you think could improve the professional lives of early-career scientists?**

Providing support for early-career scientists through research funding, family leave programs and health insurance is key. In addition, since tenure-track academic positions are not the goal for all scientists, it is important to offer career development resources and information to young scientists starting as early as college or graduate school so they can know what options they have outside of academia or within academia as a staff scientist/research associate.

**What's next for you?**

In terms of experiments, I am currently funded by a K99/R00 Pathway to Independence Award through the National Institute on Alcohol Abuse and Alcoholism (NIAAA) so my next set of experiments will investigate how prenatal alcohol exposure impacts cell cycle progression in the early gestational embryo. In addition, I am planning on entering the academic job market this year and am working to finish up a few other on-going experiments in my postdoctoral lab.

## References

[DMM049131C1] Boschen, K. E., Ptacek, T. S., Berginski, M. E., Simon, J. M. and Parnell, S. E. (2021). Transcriptomic analyses of gastrulation-stage mouse embryos with differential susceptibility to alcohol. *Dis. Model. Mech.* 14, dmm049012. 10.1242/dmm.049012 34137816 PMC8246266

